# c-Jun NH_2_-terminal kinase-dependent upregulation of DR5 mediates cooperative induction of apoptosis by perifosine and TRAIL

**DOI:** 10.1186/1476-4598-9-315

**Published:** 2010-12-20

**Authors:** Lei Fu, Yi-Dan Lin, Heath A Elrod, Ping Yue, Youtake Oh, Bo Li, Hui Tao, Georgia Z Chen, Dong M Shin, Fadlo R Khuri, Shi-Yong Sun

**Affiliations:** 1 Department of Hematology and Medical Oncology, Emory University School of Medicine and Winship Cancer Institute, Atlanta, Georgia, USA; 2 Department of Infectious Diseases, Xiangya Hosptial, Central South University, Changsha, Hunan, PR China; 3 Department of Thoracic and Cardiovascular Surgery, West China Hospital, Sichuan University, Chengdu, PR China

## Abstract

**Background:**

Perifosine, an alkylphospholipid tested in phase II clinical trials, modulates the extrinsic apoptotic pathway and cooperates with tumor necrosis factor-related apoptosis-inducing ligand (TRAIL) to augment apoptosis. The current study focuses on revealing the mechanisms by which perifosine enhances TRAIL-induced apoptosis.

**Results:**

The combination of perifosine and TRAIL was more active than each single agent alone in inducing apoptosis of head and neck squamous cell carcinoma cells and inhibiting the growth of xenografts. Interestingly, perifosine primarily increased cell surface levels of DR5 although it elevated the expression of both DR4 and DR5. Blockade of DR5, but not DR4 upregulation, via small interfering RNA (siRNA) inhibited perifosine/TRAIL-induced apoptosis. Perifosine increased phosphorylated c-Jun NH_2_-terminal kinase (JNK) and c-Jun levels, which were paralleled with DR4 and DR5 induction. However, only DR5 upregulaiton induced by perifosine could be abrogated by both the JNK inhibitor SP600125 and JNK siRNA. The antioxidants, N-acetylcysteine and glutathione, but not vitamin C or tiron, inhibited perifosine-induced elevation of p-c-Jun, DR4 and DR5. Moreover, no increased production of reactive oxygen species was detected in perifosine-treated cells although reduced levels of intracellular GSH were measured.

**Conclusions:**

DR5 induction plays a critical role in mediating perifosine/TRAIL-induced apoptosis. Perifosine induces DR5 expression through a JNK-dependent mechanism independent of reactive oxygen species.

## Background

Perifosine, the first orally bioavailable alkylphospholipid, has shown antitumor activity in preclinical models and is currently in Phase II clinical trials [1,2]. The mechanisms underlying perifosine-mediated antitumor effects have not been fully elucidated, although it is known to inhibit Akt [3,4] and induce c-Jun NH_2_-terminal kinase (JNK) activation [5-7]. Perifosine has also been shown to induce p21 expression, leading to cell cycle arrest [8]. In addition, perifosine in combination with other antitumor agents such as the PDK1 inhibitor, UCN-01 [9], histone deacetylase inhibitors [10], and the chemotherapeutic agent etoposide [11] show synergistic antitumor effects.

Tumor necrosis factor (TNF)-related apoptosis-inducing ligand (TRAIL; also called APO-2L), a member of the TNF family, induces apoptosis preferentially in transformed or malignant cells, thus making it distinct from the death ligands TNFα and Fas, which, in addition to inducing apoptosis in cancer cells, cause an inflammatory response and liver damage, respectively, when administered systemically [12,13]. Therefore, TRAIL is currently being tested in phase I oncology trials as a tumor-selective apoptosis-inducing cytokine.

Perifosine was previously reported to be active in inhibiting the growth of head and neck squamous cell carcinoma (HNSCC) cells [8]. However, a phase II trial of perifosine in recurrent or metastatic head and neck cancer failed to demonstrate the single-agent activity of perifosine in HNSCC [14]. Therefore, we are interested in developing perifosine-based combinations that exert augmented anticancer efficacy. Our previous studies have shown that perifosine increases DR5 expression and cooperates with TRAIL to augment apoptosis in human lung cancer and myeloma cells [15,16]. The current study validated the cooperative induction of apoptosis by perifosine and TRAIL in human HNSCC cells and examined their combinatorial effect on the growth of HNSCC xenografts. Importantly, we were particularly interested in revealing the possible mechanisms underlying death receptor induction by perifosine and the cooperative induction of apoptosis by the perifosine/TRAIL combination.

## Methods

### Reagents

Perifosine was supplied by Keryx Biopharmaceuticals, Inc (New York, NY). This agent was dissolved in PBS and stored at -20°C. Stock solution was diluted to the appropriate concentrations with growth medium immediately before use. Human recombinant TRAIL used in cell cultures and in animals was purchased from PeproTech, Inc. (Rocky Hill, NJ) and prepared as previously described [17]. The specific JNK inhibitor SP600125 was purchased from Biomol (Plymouth Meeting, PA). 2'7'-dichlorofluorescein diacetate (DCF-DA) was purchased from Molecular Probes (Eugene, OR). Mouse anti-caspase-3 monoclonal antibody was purchased from Imgenex (San Diego, CA). Rabbit polyclonal antibodies against p-c-Jun (Ser63), c-Jun, p-ERK1/2 (Thr202/Tyr204), ERK1/2, p-p38 (Thr180/Tyr182), p38, caspase-8, caspase-9, and poly(ADP-ribose) polymerase (PARP) were purchased from Cell Signaling Technology (Beverly, MA). Rabbit polyclonal anti-DR5 antibody was purchased from ProSci Inc (Poway, CA). Mouse monoclonal anti-DR4 antibody (B-N28) was purchased from Diaclone (Stamford, CT). Rabbit anti-β-actin polyclonal antibody and other chemicals were purchased from Sigma Chemicals (St. Louis, MO).

### Cell Lines and Cell Culture

The cell lines used in this study (M4e, 22A and 1483) were described previously [18,19] and cultured in Dulbecco's modified Eagle's medium (DMEM)/F12 supplemented with 5% fetal bovine serum.

### Cell Viability Assay

Cells were cultured in 96-well cell culture plates and treated the next day with the agents indicated. Viable cell numbers were estimated using the sulforhodamine B (SRB) assay, as previously described [20].

### Colony Formation Assay

Colony formation on plate was conducted in 12-well cell culture plates as previously described [21].

### Western Blot Analysis

Preparation of whole cell protein lysates and Western blot analysis were described previously [22,23].

### Detection of Caspase Activation and Apoptosis

Caspase activation and their substrate cleavage were detected by Western blot analysis as described above. Apoptosis was detected by estimating sub-G1 population [24] or by measuring Annexin V positive cell numbers with Annexin V-phycoerythrin (PE) apoptosis detection kit purchased from BD Biosciences (San Jose, CA), following the manufacturer's instructions.

### Detection of Intracellular Reactive Oxygen Species (ROS) and Glutathione (GSH)

Intracellular ROS generation was detected using the oxidation-sensitive fluorescent dye DCF-DA as previously described [25]. The total intracellular GSH levels were measured using monochlorobimane as a probe, as previously described [26].

### Detection of DR4 and DR5 mRNA Levels

Total cellular RNA isolation, cDNA synthesis and DR5 amplification were the same as described previously [27]. DR4 and GAPDH were amplified by PCR using the following primers: DR4 sense, 5'-CAGAGGGATGGTCAAGGTCAAGG-3'; DR4 antisense, 5'-CCACAACCTGAGCCGATGC-3'; GAPDH sense, 5'-TGATGACATCAAGAAGGTGGTGAAG-3'; and GAPDH antisense, 5'-TCCTTGGAGGCCATGTGGGCCAT-3'. For DR4 amplification the 20 μL amplification mixture contained 1 μL of cDNA, 0.6 μL of MgCl_2 _(50 mM), 1 μL each of the sense and antisense primers (20 μM each), 0.4 μL of dNTP (10 mM), 1 μL of iTaq DNA Polymerase (25 units/μL; Bio-Rad Laboratories), 2 μL 10 × reaction buffer, and sterile H_2_O. PCR was done for 26 cycles. After an initial step at 95°C for 3 minutes, each cycle consisted of 30 sec of denaturation at 95°C, 30 sec of annealing at 58°C, and 45 sec of extension at 72°C. This was followed by an additional extension step at 72°C for 7 min. The housekeeping gene GAPDH was also amplified as an internal reference. PCR products were resolved by electrophoresis on a 1.0% agarose gel, stained, and directly visualized under UV illumination.

### Detection of Cell Surface DR4 and DR5

The procedure for direct antibody staining and subsequent flow cytometric analysis of cell surface protein was described previously [28,29]. The mean fluorescence intensity (MFI) that represents antigenic density on a per cell basis was used to represent cell surface DR5 or DR4 expression level. PE-conjugated mouse anti-human DR5 (DJR2-4) and anti-human DR4 (DJR1) monoclonal antibodies and PE mouse IgG1 isotype control (MOPC-21/P3) were purchased from eBioscience (San Diego, CA).

### Small Interfering RNA (siRNA)-mediated Gene Silencing

High purity control (non-silencing) and DR5 siRNA oligos were described previously [22]. DR4 siRNA targets the sequences 5'-AATGAGATCGATGTGGTCAGA-3' (1239-1259). These siRNA oligos were synthesized from Qiagen (Valencia, CA). JNK siRNAs (Cat No. 6234) were purchased from Cell Signaling Technology. Transfection of these siRNA duplexes was conducted in 6-well plates using the HiPerFect transfection reagent (Qiagen) following the manufacturer's manual. Forty-eight hours later, the cells were treated with or without the combination of perifosine and TRAIL. Gene silencing effect was evaluated by Western blot analysis.

### HNSCC Orthotopic Xenograft Mouse Model

The animal experiment was approved by the Animal Care and Use Committee of Emory University. Nude mice (athymic nu/nu, Taconic, NY, USA) aged 4-6 weeks were randomized into 4 groups. Each mouse was injected with 1 × 10^6 ^M4e cells in 100 μl of PBS into the submandibular to mylohyoid muscle as described previously [30]. After about one week, the mice received the following treatments: vehicle control, perifosine (20 mg/kg; og), TRAIL (20 mg/kg; ip) and perifosine plus TRAIL for three weeks (once a day, 5 days of treatment plus two days of break per week). The mice were then sacrificed and the tumors were removed and weighed.

## Results

### Perifosine Cooperates with TRAIL to Augment Induction of Apoptosis, Reduce Colony Formation and Inhibit the Growth of HNSCC Xenografts

**Figure 1:**
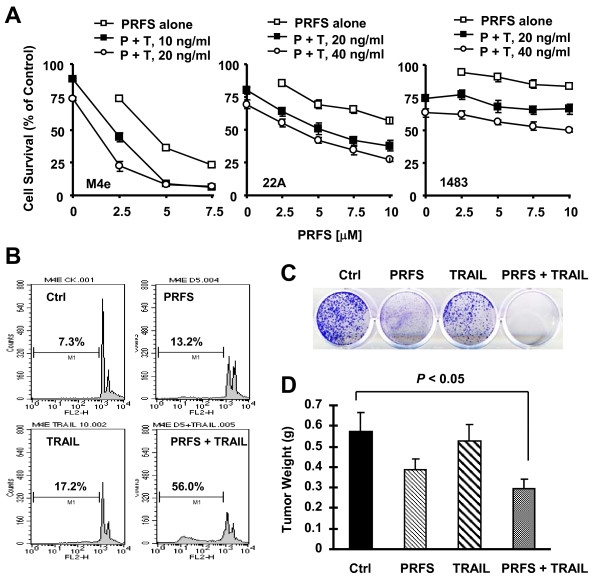
**The combination of perifosine and TRAIL exhibits enhanced effects on decreasing cell survival (*A*), inducing apoptosis (*B*) and inhibiting colony formation (*C*) in HNSCC cell lines and augments xenograft growth inhibition (*D*)**. *A*, The indicated cell lines were seeded in 96-well plates and the next day treated with the indicated concentrations of TRAIL alone, perifosine (PRFS) alone, or their respective combinations (P + T). After 24 h, the cells were subjected to estimation of cell number using the SRB assay. The data are means ± SD of four replicate determinations. *B*, M4e cells were treated with PBS control, 5 μM perifosine, 10 ng/ml TRAIL and the combination of perifosine and TRAIL. After 24 h, apoptotic (sub-G1) cells were measured by flow cytometry. *C*, M4e cells plated in 12-well cell culture plates were treated with 1 μM perifosine, 10 ng/ml TRAIL, or their combination. The same treatments were repeated every 3 days. After 12 days, the plates were stained for cell colonies with crystal violet dye, and photographs of colonies taken using a digital camera. *D*, Four groups of mice were treated with PBS (n = 11), perifosine (n = 11), TRAIL (n = 13) and perifosine combined with TRAIL (n = 13), respectively, as described in "Materials and Methods". After about 3 weeks, the tumors were removed from the sacrificed mice and weighed. The data are means ± SD.

We first determined whether perifosine combined with TRAIL augmented the induction of apoptosis in HNSCC cell lines. As presented in Figure 1A, the combination of perifosine and TRAIL was more potent than each single agent alone in decreasing the survival of M4e and 22A cells. For example, perifosine at 2.5 μM alone and TRAIL at 20 ng/ml alone decreased the survival of M4e cells by less than 25%, whereas their combination decreased cell survival by greater than 75%. This enhanced effect was minimal in 1483 cells. By measuring apoptotic cells, we detected 56% apoptotic cells in M4e cells exposed to the combination of perifosine and TRAIL and <20% apoptotic cells in M4e cells treated with either perifosine or TRAIL alone (Figure 1B). This result further demonstrates that the combination of perifosine and TRAIL exhibits a more than additive (i.e., synergistic) effect on induction of apoptosis. Thus, we conclude that perifosine cooperates with TRAIL to synergistically trigger apoptosis of HNSCC cells.

Moreover, we analyzed the long-term effect of the combination of perifosine and TRAIL on clonogenic survival in cell culture and xenograft growth in nude mice. In agreement with the apoptosis study, the combination of perifosine and TRAIL was much more potent than either agent alone in suppressing colony formation. Specifically the combination almost eliminated all colonies, whereas perifosine or TRAIL alone only partially inhibited the formation and growth of colonies (Figure 1C). Under the tested experimental conditions, we found that the combination, but not perifosine alone or TRAIL alone, also significantly inhibited the growth of M4e xenografts (*P *< 0.05, Figure 1D). Thus, the combination of perifosine and TRAIL exhibits an enhanced tumor-inhibitory effect *in vivo*.

### Perifosine Upregulates the Expression of DR4 and DR5

**Figure 2:**
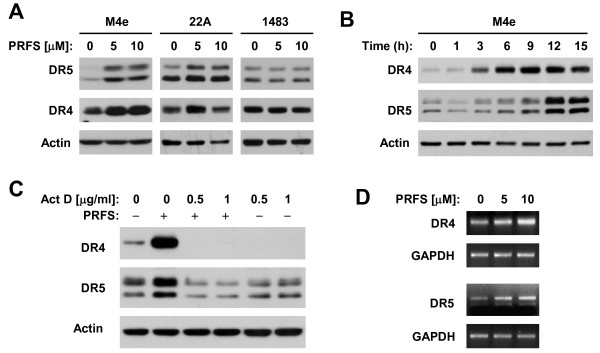
**Perifosine induces DR4 and DR5 expression (*A *and *B*) at the transcriptional level (*C *and *D*)**. *A *and *B*, The indicated cell lines were treated with the given concentrations of perifosine (PRFS) for 16 h. *C*, M4e cells were pre-treated with the given concentrations of Act D for 30 min and then co-treated with 10 μM perifosine for 8 h. After the aforementioned treatments, the cells were subjected to preparation of whole-cell protein lysates and subsequent detection of the indicated proteins using Western blot analysis. *D*, M4e cells were treated with indicated concentrations of perifosine for 7 h and then subjected to preparation of cellular total RNA and subsequent RT-PCR for detection of DR4, DR5, and GAPDH (internal loading control).

To investigate how perifosine cooperates with TRAIL to augment apoptosis, we examined the effects of perifosine on the expression of DR4 and DR5, which are known to be TRAIL death receptors. As presented in Figure 2A, both DR4 and DR5 were substantially increased by perifosine in both M4e and 22A cell lines, in which the perifosine and TRAIL combination exerted augmented cell death-inducing effects, but not in 1483 cells, in which the combination did not exhibit an enhanced cell death effect. In M4e cells, we further conducted time-course analyses of the changes in expression of DR4 and DR5. As presented in Figure 2B, upregulation of both DR4 and DR5 levels occurred at 3 h post perifosine treatment and was sustained for up to 15 h. Collectively, these results indicate that the upregulation of DR4 and DR5 by perifosine is an early event that may contribute to cooperative induction of apoptosis by the perifosine and TRAIL combination.

### Induction of DR5, but not DR4, Plays a Critical Role in Mediating Cooperative Induction of Apoptosis by Perifosine and TRAIL Combination

**Figure 3:**
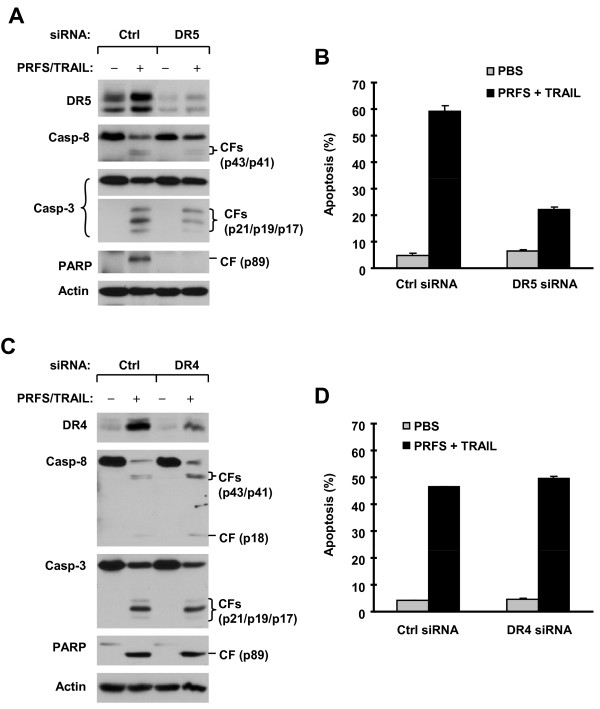
**siRNA-mediated inhibition of perifosine-induced upregulation of DR5 (*A*), but not DR4 (*B*), attenuates augmented induction of apoptosis by the combination of perifosine and TRAIL**. M4e cells were transfected with control (Ctrl), DR5 (A) or DR4 (B) siRNA. After 48 h, the cells were treated without and with the combination of 5 μM perifosine and 12.5 ng/ml TRAIL (PRFS/TRAIL). After 24 h, the cells were harvested for preparation of whole-cell protein lysates and subsequent Western blot analysis to detect DR5, DR4 and caspase cleavage (*A *and *C*) and for detection of apoptosis with annexin V-flow cytometry (*B *and *D*). Columns in *B *and *C *are means ± SD of duplicated determinations.

To dissect the roles of DR4 and DR5 modulation in mediating perifosine/TRAIL-induced apoptosis, we used a siRNA approach to block DR4 or DR5 induction through silencing their expression and then examined the impact on induction of apoptosis by the combination of perifosine and TRAIL. As shown in Figure 3, transfection of DR4 or DR5 siRNA blocked perifosine-induced upregulation of DR4 or DR5, indicating the successful blockade of DR4 or DR5 induction, respectively (Figures 3A and 3C). The combination of perifosine and TRAIL increased the levels of cleaved caspase-8, caspase-3 and PARP (Figure 3A) and apoptotic populations (Figure 3B) in control siRNA-transfected cells. However, these effects of the combination were clearly attenuated in cells transfected with DR5 siRNA (Figures 3A and 3B). In contrast, blockade of DR4 induction exhibited no protective effects on the cleavage of caspases and PARP and induction of apoptosis (Figures 3C and 3D) induced by the perifosine and TRAIL combination when compared with control siRNA-transfected cells. Thus, DR5 induction, but not DR4 upregulation, plays an important role in mediating perifosine/TRAIL-induced apoptosis.

### Perifosine Increases Cell Surface Levels of DR5, but not DR4

**Figure 4:**
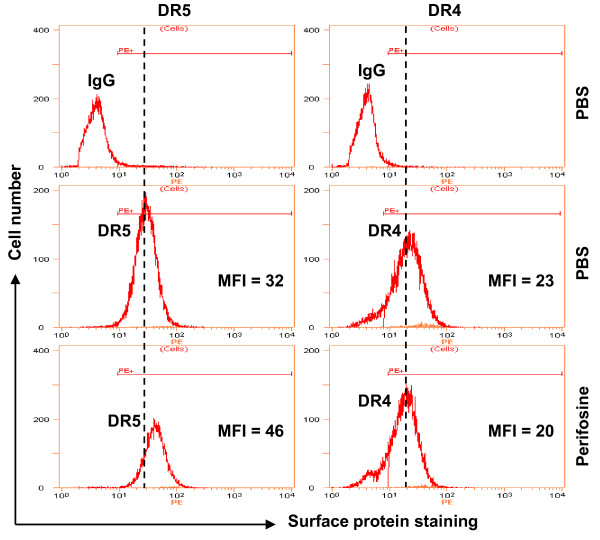
**Perifosine increases cell surface levels of DR5 (*A*), but not DR4 (*B*), in M4e cells**. M4e cells were treated with PBS or 10 μM perifosine for 12 h and then harvested for analysis of cell surface DR5 and DR4 by immunofluorescent staining and subsequent flow cytometry.

To further demonstrate the roles of DR4 and DR5 upregulation in mediating perifosine/TRAIL-induced apoptosis, we determined whether perifosine increases cell surface levels of DR4 and/or DR5 with flow cytometry. As presented in Figure 4, the MFIs for DR5 in PBS control and periforsine-treated cells were 32 and 46, respectively, indicating that perifosine increases cell surface levels of DR5. However, perifosine did not increase cell surface levels of DR4 because the MFIs for DR4 in PBS control and periforsine-treated cells were 23 and 20, respectively. Thus, it appears that perifosine specifically increases cell surface levels of DR5 in the tested cell system.

### Perifosine Induces DR4 and DR5 Expression at the Transcriptional Level

Considering the critical role of DR5 induction in mediating the cooperative induction of apoptosis by perifosine and TRAIL as documented above, we then focused our further study on understanding how perifosine induces DR5 expression in comparison with DR4 upregulation. Thus, we asked whether perifosine regulates the expression of DR5 and DR4 at the transcriptional level. To this end, we first examined the effects of perifosine on DR5 and DR4 expression in the presence of the transcriptional inhibitor actinomycin D (Act D). As shown in Figure 2C, perifosine increased the levels of DR5 and DR4 in the absence, but not in the presence, of Act D, indicating that inhibition of transcription abolishes perifosine's ability to increase DR5 and DR4 expression. Furthermore, we directly examined whether perifosine increased the mRNA levels of DR5 and DR4. As shown in Figure 2D, we detected dose-dependent increases in DR5 and DR4 mRNAs in cells exposed to perifosine. Collectively, we conclude that perifosine increases the expression of DR5 and DR4 at the transcriptional level.

### Perifosine Induces JNK-dependent Expression of DR5

**Figure 5:**
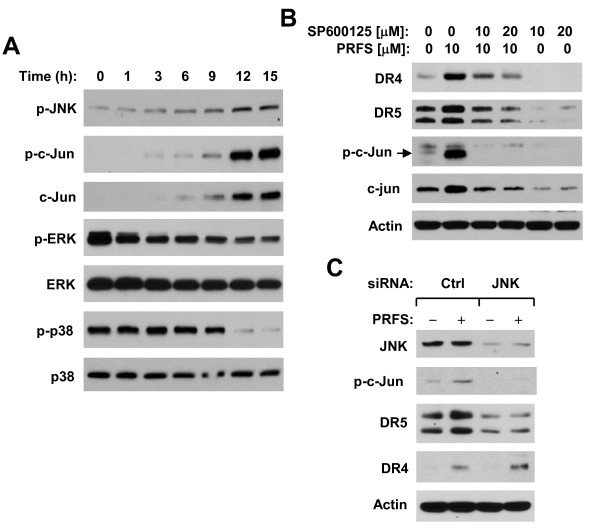
**Perifosine activates JNK signaling (*A*), which mediates DR5 induction by perifosine (*B *and *C*)**. *A*, M4e cells were treated with 10 μM perifosine for the times indicated. *B*, M4e cells were pre-treated with the given concentrations of SP600125 for 30 min and then co-treated with 10 μM perifosine for 12 h. *C*, M4e cells were transfected with control (Ctrl) or JNK siRNA for 48 h and then treated with PBS or perifosine for additional 12 h. After the aforementioned treatments, the cells were subjected to preparation of whole-cell protein lysates and subsequent detection of the indicated proteins using Western blot analysis.

In addition to Akt inhibition, perifosine modulates other signaling pathways such as ERK, p38 and JNK [7,31]. In our cell system, perifosine rapidly increased the levels of p-JNK and p-c-Jun while decreasing the levels of p-ERK1/2 and p-p38 (Figure 5A), indicating that perifosine activates JNK and suppresses ERK and p38 signaling pathways. These results are consistent with a previous report [7]. Given the drastic induction of DR5 and DR4 by perifosine as demonstrated above, we were particularly interested in the mechanisms by which perifosine induces expression of DR5 and DR4. In the same cell system exposed to perifosine, we noted that both DR4 and DR5 induction were kinetically paralleled with p-c-Jun increase, both of which occurred after 3 h treatment and reached a peak at 12 h treatment (Figures 2B and 5A). Since JNK activation is linked to DR5 induction as we previously demonstrated [32], we then asked whether perifosine induces DR5 and DR4 expression via a JNK-dependent mechanism. To this end, we examined the effect of the JNK inhibitor SP600125 on perifosine-induced DR4 and DR5 expression. The presence of SP600125 not only decreased basal levels of p-c-Jun, DR4 and DR5, but also blocked perifosine-induced increases in p-c-Jun, DR4 and DR5 expression (Figure 5B). To robustly demonstrate the role of JNK activation in mediating perifosine-induced DR4 and DR5 expression, we transfected JNK-specific siRNA to inhibit JNK activation through silencing JNK expression and then examined its impact on DR4 and DR5 expression. As presented in Figure 5C, transfection of JNK siRNA reduced basal levels of JNK and p-c-Jun and abrogated perifosine-induced p-c-Jun increase, indicating the successful knockdown of JNK and inhibition of JNK activity. As expected, perifosine induced DR5 expression in control siRNA-transfected cells, but not in JNK siRNA-transfected cells. We noted that knockdown of JNK also reduced basal levels of DR5. Unexpectedly, we found that knockdown of JNK did not inhibit perifosine's ability to increase DR4 expression (Figure 5C). Collectively, we conclude that perifosine induces DR5 expression through a JNK-dependent mechanism.

### The Thiol Antioxidants N-acetylcysteine (NAC) and GSH Inhibit Perifosine-induced JNK Activation and Upregulation of DR4 and DR5

**Figure 6:**
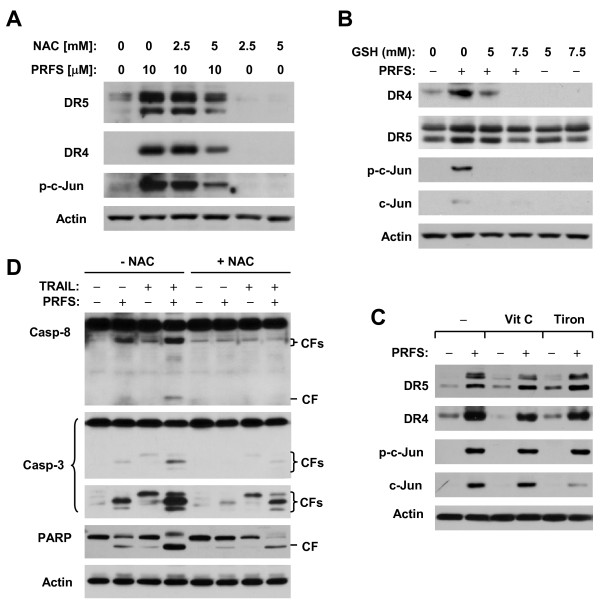
**Effects of NAC and other antioxidants on perifosine-induced DR4 and DR5 expression (*A*-C) and on enhancement of TRAIL-induced apoptosis by perifosine (*D*)**. *A*, M4e cells were pre-treated with the given concentrations of NAC for 30 min and then co-treated with 10 μM perifosine for 12 h; *B*, M4e cells were pre-treated with 0.5 mM Vitamin C (Vit C), or 0.5 mM tiron for 30 min and then co-treated with 10 μM perifosine for 10 h; *C*, M4e cells were pre-treated with the given concentrations of GSH for 30 min and then co-treated with 10 μM perifosine for 10 h. *D*, M4e cells were pre-treated with 5 mM NAC for 30 min and co-treated with 5 μM perifosine, 12.5 ng/ml and the combination of perifosine and TRAIL for an additional 12 h. After the aforementioned treatments, the cells were subjected to preparation of whole-cell protein lysates and subsequent detection of the indicated proteins using Western blot analysis.

It was suggested that ROS production is involved in mediating perifosine-induced DR5 expression [33]. Thus, we further determined whether this is also the mechanism underlying perifosine-induced DR4 and DR5 upregulation in our cell systems. In the presence of a high concentration of NAC (e.g., 5 mM), perifosine-induced increases in p-c-Jun, DR4 and DR5 were attenuated (Figure 6A), as was perifosine/TRAIL-induced apoptosis, as evidenced by reduced levels of cleaved caspase-8, caspase-3 and PARP in the cells co-treated with NAC (Figure 6D). In addition, we further examined the effects of other antioxidants besides NAC on perifosine-induced JNK activation and expression of DR4 and DR5, since we assumed that these antioxidants should also be able to inhibit perifosine-induced JNK activation and expression of DR4 and DR5 if ROS indeed plays a role in this process. As presented in Figures 6B and 6C, we found that GSH, but not vitamin C or tiron, blocked perifosine-induced increases in p-c-Jun, DR4 and DR5 to the same extent as NAC. Thus, it appears that only thiol antioxidants such as NAC and GSH block perifosine-induced JNK activation and upregulation of DR4 and DR5.

### Perifosine Reduces Total Intracellular Levels of GSH Without Increasing ROS Generation

**Figure 7:**
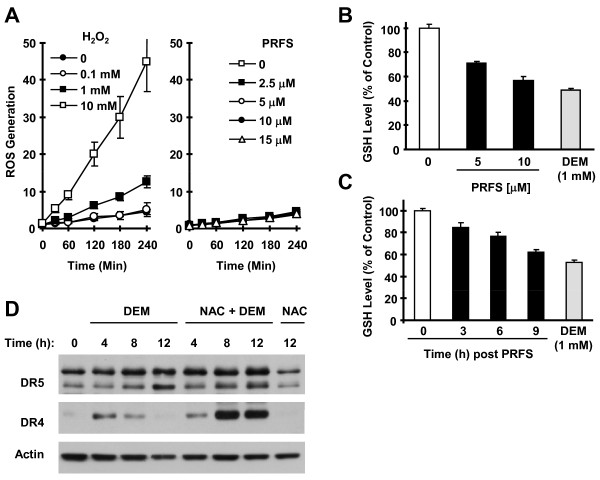
**Effects of perifosine on ROS generation (*A*) and GSH levels (*B *and *C*) and effects of DEM on DR4 and DR5 expression in the absence and presence of NAC (*D*)**. *A*, M4e cells were loaded with DCF-DA and then exposed to the indicated concentrations of perifosine (PRFS) or H_2_O_2 _(positive control). At the indicated times post addition of the agents, ROS generation was monitored as described in "Materials and Methods". *B *and *C*, M4e cells were treated with the indicated concentrations of perifosine or DEM (*B*) for 9 h or treated with 10 uM for the given times or 1 mM DEM for 9 h (*C*). The cells were then subjected to GSH assay as described in "Materials and Methods". *D*, M4e cells were treated with 1 mM DEM in the absence and presence of 5 mM NAC for the indicated times (NAC was added to the cells 30 min before addition of perifosine). The cells were then lysed for preparation of whole-cell protein lysates and subsequent detection of the indicated proteins using Western blot analysis.

We then determined whether perifosine indeed stimulates ROS generation in our cells. Unexpectedly, we failed to detect increased ROS production in perifosine-treated cells, while H_2_O_2_, as a positive control, substantially increased ROS generation (Figure 7A). Thus, perifosine does not initiate ROS generation in our cell system. Because of the unique effects of NAC and GSH on blockage of perifosine-induced JNK activation and DR4 and DR5 upregulation, we further determined whether perifosine decreases intracellular GSH levels. By analyzing the intracellular GSH content in M4e cells treated with perifosine, we detected decreased levels of GSH in both time- and dose-dependent manners after exposure to perifosine (Figures 7B and 7C). Diethylmaleate (DEM), a positive control known to be a GSH-depleting agent [34], also decreased GSH content. Thus, perifosine reduces intracellular GSH levels.

### DEM Weakly Induces DR4 and DR5 Expression, Which Can Be Enhanced by NAC

If reduction of GSH levels is sufficient to result in upregulation of DR4 and DR5, we assumed that DEM should induce DR4 and DR5 expression through a similar mechanism as perifosine does. Thus, we examined the effects of DEM in the absence and presence of NAC on DR4 and DR5 expression. As presented in Figure 7D, DEM weakly increased the levels of DR4 and DR5 in M4e cells. We noted that DR4 induction was transient, since it increased at 4 h and declined at 8 h and particularly at 12 h. The presence of NAC did not inhibit the induction of DR4 and DR5; instead it enhanced and sustained the expression of DR4 and DR5 induced by DEM. These data suggest that DEM induces DR4 and DR5 through a different mechanism from perifosine.

## Discussion

In agreement with findings in other cell types, we have demonstrated that perifosine in combination with TRAIL exhibits enhanced apoptosis-inducing activity in HNSCC cells. Moreover, we have shown that the combination of perifosine and TRAIL exhibits enhanced effects on decreasing clonogenic survival and on inhibiting the growth of HNSCC xenograft in an orthotopic mouse model (Figure 1). Thus the current results warrant further evaluation of the perifosine and TRAIL combination as a potential therapeutic regimen against HNSCC. We noted that the perifosine and TRAIL combination was more effective in decreasing cell survival in M4e and 22A cells than in 1483 cells, indicating that cell lines have different sensitivity to the combination. Thus it is important to fully understand how perifosine cooperates with TRAIL to augment apoptosis so that we can predict cell sensitivity to the combination of perifosine and TRAIL.

In this study, we found that perifosine increased the expression of DR4 and DR5 in both M4e and 22A cells, but not in 1483 cells, suggesting the possible involvement of the upregulation of these proteins in perifosine-mediated augmentation of TRAIL-induced apoptosis because both proteins are receptors for TRAIL. Although both DR4 and DR5 upregulation are early events in cells exposed to perifosine, gene silencing-mediated blockade of DR5 induction, but not DR4 induction, attenuated apoptosis induced by the perifosine and TRAIL combination (Figure 3), indicating that DR5 upregulation is a more critical event than DR4 induction for perifosine to augment TRAIL-induced apoptosis. This finding raised the interesting question of why DR4 is not involved in modulation of perifosine/TRAIL-induced apoptosis since it is also upregulated by perifosine. By analyzing cell surface DR4 and DR5, we found that perifosine increased cell surface levels of DR5, but not DR4 (Figure 4). This may, at least in part, explain why upregulation of DR5, but not DR4, is critical for mediating augmentation of TRAIL-induced apoptosis by perifosine.

DR4 and DR5 induction by perifosine was reported previously by our group [15,16] and others [33]. However, how perifosine upregulates DR4 and DR5 has not been fully elucidated. In this study, we found that Act D abolished perifosine's ability to upregulate DR4 and DR5 expression. Moreover, we detected increased levels of DR4 and DR5 mRNA in cells exposed to perifosine (Figure 2). These data together indicate that perifosine increases the expression of DR4 and DR5 at the transcriptional level. It has been previously shown that JNK activation positively regulates DR5 and DR4 expression induced by certain drugs [32,35,36]. It has also been documented that perifosine activates JNK signaling [5-7,37]. A recent study by Tazzari et al. [33] suggests that perifosine induces a JNK-dependent DR5 expression in leukemia cells. In our study, we found that perifosine increased the levels of p-JNK and p-c-Jun, indicating that perifosine also activates JNK signaling. We noted that JNK signaling activation paralleled the upregulation of DR4 and DR5. In the presence of the JNK inhibitor SP600125, perifosine-induced upregulation of both DR4 and DR5 was inhibited (Figure 5). Interestingly, inhibition of JNK with knockdown of JNK expression abolished perifosine-induced upregulation of DR5, but not DR4 (Figure 5C). Thus, it is clear that perifosine induces JNK-dependent DR5 expression. Meanwhile, these data also suggest that JNK activation is not sufficient for perifosine to induce DR4 expression. Considering the specificity of small molecule inhibitors, it is important to validate results generated using a small molecule inhibitor with a specific molecular approach (e.g., siRNA).

A previous study of leukemia cells has shown that perifosine induces ROS generation, which contributes to perifosine-induced DR5 expression. This study primarily used NAC as the only antioxidant to demonstrate the involvement of ROS in perifosine-induced DR5 expression [33]. In agreement, we found that NAC at high concentrations (e.g., 5 mM) attenuated perifosine's ability to increase DR4 and DR5 expression and to augment TRAIL-induced apoptosis (Figures 6A and 6B). However, we failed to detect increased ROS generation in cells exposed to perifosine (Figure 6C). After utilization of additional antioxidents, we observed that another thiol antioxidant, GSH, could also prevent DR4 and DR5 induction by perifosine, but other two non-thiol antioxidants, vitamin C and tiron, could not (Figure 6D). These data thus argue against the involvement of ROS in mediating induction of DR4 and DR5 by perifosine, at least in our cell system. We noted that both NAC and GSH blocked perifosine-induced JNK activation and DR4 and DR5 upregulation, whereas vitamin C and tiron, which did not inhibit perifosine-induced DR4 and DR5 expression, did not affect perifosine-induced p-c-Jun increase (Figure 6). Therefore, it appears that JNK activation, but not ROS generation, plays an essential role in mediating DR5 upregulation by perifosine.

NAC is an aminothiol and synthetic precursor of intracellular cysteine and GSH and is thus considered an important antioxidant; however, NAC also possesses a reducing property through its thiol-disulfide exchange activity [38]. There are precedents that NAC protects drug-induced apoptosis through its thiol-disulfide exchange activity independent of its antioxidant activity [26,38,39]. In our study, we found that perifosine decreased the levels of intracellular GSH, as did DEM. Similarly, a recent study by Simons et al [40] reported that perifosine increases oxidized levels of GSH and glutathione disulfide (GSSG), and that its combination with a glutathione-inhibiting agent enhances perifosine's cell killing effects in HNSCC cells. It is known that DEM forms a covalent adduct with GSH via a reaction catalyzed by glutathione-S-transferase, leading to depletion of intracellular GSH [34]. In our study, DEM weakly increased DR4 and DR5 expression; which was further enhanced rather than inhibited by NAC (Figure 7D), suggesting that perifosine and DEM have different mechanisms of regulating DR4 and DR5. These findings also suggest that simple reduction of intracellular GSH is not sufficient to induce substantial upregulation of DR4 and DR5. It is possible that perifosine may act directly on the sulfhydryl (-SH) group of cellular components or proteins as other agents do [38], activating the JNK signaling pathway as well as other mechanisms and subsequent upregulation of DR5 and DR4. It is also possible that perifosine activates JNK signaling through an unknown mechanism, which can be enhanced by reduction of GSH. Thiol antioxidants may directly interact with perifosine or prevent the reduction of GSH, leading to abolishing or attenuating perifosine's ability to activate JNK and induce DR5 expression. It is known that glutathione S-transferases (GSTs) inhibit JNK activity by directly interacting with JNK [41-44]. Moreover, GSH has been shown to inhibit JNK activity, likely through affecting the GST-JNK interaction [44]. It is possible that perifosine directly interacts with sulfhydryl group of GSTs, releasing GST from its interaction with JNK and eventually activating JNK. Reduction of cellular GSH will further enhance this process. Nonetheless, future studies are needed to demonstrate the potential role of GSTs in perifosine-induced JNK activation.

It has been suggested that Akt negatively regulates the JNK signaling pathway [45]. Given that perifosine has potent Akt-inhibitory activity, it is also possible that perifosine activates JNK signaling through the release of Akt-mediated JNK inhibition by inhibiting AKT. We tested the effect of another Akt inhibitor called API-1 [46] on JNK activation in the same cell system and found that API-1 also increased p-c-Jun levels (data not shown). Thus, further study in this direction is also warranted.

## Conclusions

The current study has demonstrated that perifosine induces a JNK-dependent DR5 upregulation independent of ROS generation. Although perifosine induces expression of both DR4 and DR5, DR5, but not DR4, induction is critical for cooperative augmentation of apoptosis by perifosine and TRAIL.

## Abbreviations

Act D: actinimycin D; DEM: diethylmaleate; DR: death receptor; GSH: glutathione; HNSCC: head and neck squamous cell carcinoma; JNK: c-Jun NH_2_-terminal kinase; NAC: N-acetylcysteine; ROS: reactive oxygen species; siRNA: small interfering RNA; TRAIL: tumor necrosis factor-related apoptosis-inducing ligand.

## Competing interests

The authors declare that they have no competing interests.

## Authors' contributions

LF, YDL HAE, PY, YTO, BL and HT designed and conducted experiments and data analysis. GZC provided cell lines and helped with animal study. DMS and FRK participated in discussion of the data. SYS participated in experimental design, coordination, data analysis and draft of the manuscript. All authors read and approved the final manuscript.
